# Efficacy and safety of EGFR-TKI combined with WBRT vs. WBRT alone in the treatment of brain metastases from NSCLC: a systematic review and meta-analysis

**DOI:** 10.3389/fneur.2024.1362061

**Published:** 2024-04-26

**Authors:** Shuai Li, Shumei Xu, Luwei Li, Zhihong Xue, Lang He

**Affiliations:** ^1^Chengdu University of Traditional Chinese Medicine, Chengdu, Sichuan, China; ^2^Cancer Prevention and Treatment Institute of Chengdu, Department of Oncology, Chengdu Fifth People’s Hospital (The Second Clinical Medical College), Affiliated Fifth People’s Hospital of Chengdu University of Traditional Chinese Medicine, Chengdu, Sichuan, China

**Keywords:** EGFR-TKI, WBRT, non-small lung cancer, brain metastase, RCT

## Abstract

**Background:**

The efficacy and safety of combining epidermal growth factor receptor tyrosine kinase inhibitors (EGFR-TKIs) with whole-brain radiotherapy (WBRT) for treating brain metastases in non-small cell lung cancer patients remains to be determined.

**Methods:**

A systematic search was conducted using databases including PubMed, Embase, Web of Science, Cochrane, Wanfang, and China National Knowledge Infrastructure (CNKI), aiming to identify relevant clinical studies on the treatment of brain metastases originating from non-small cell lung cancer through the combination of EGFR-TKI and WBRT. Statistical analysis was performed utilizing Stata 17.0 software, covering clinical studies published until March 1, 2023.

**Results:**

This analysis incorporated 23 randomized controlled trials (RCTs), involving a total of 2,025 patients. Of these, 1,011 were allocated to the group receiving both EGFR-TKI and WBRT, while 1,014 were assigned to the WBRT alone group. The findings reveal that the combination of EGFR-TKI and WBRT significantly improves the intracranial objective remission rate (RR = 1.57, 95% CI: 1.42–1.74, *p* < 0.001), increases the intracranial disease control rate (RR = 1.30, 95% CI: 1.23–1.37, *p* < 0.001), and enhances the 1-year survival rate (RR = 1.48, 95% CI: 1.26–1.73, *p* < 0.001). Additionally, this combined treatment was associated with a significant survival advantage (RR = 1.48, 95% CI: 1.26–1.73, *p* < 0.001) and a reduced incidence of adverse effects (RR = 0.65, 95% CI: 0.51–0.83, *p* < 0.001), particularly with respect to nausea and vomiting (RR = 0.54, 95% CI: 0.37–0.81, *p* = 0.002) and myelosuppression (RR = 0.59, 95% CI: 0.40–0.87, *p* = 0.008). However, no statistically significant differences were observed for diarrhea (RR = 1.15, 95% CI: 0.82–1.62, *p* = 0.418), and skin rash (RR = 1.35, 95% CI: 0.88–2.07, *p* = 0.164).

**Conclusion:**

In contrast to WBRT alone, the combination of EGFR-TKI and WBRT significantly improves intracranial response, enhancing the objective response rate, disease control rate, and 1-year survival rate in NSCLC patients with brain metastases. Moreover, aside from mild cases of rash and diarrhea, there is no statistically significant increase in the incidence of additional adverse effects. Based on the comprehensive evidence collected, the use of third-generation EGFR-TKI combined with WBRT is recommended as the preferred treatment for NSCLC patients with brain metastases, offering superior management of metastatic brain lesions.

**Systematic review registration:**

https://www.crd.york.ac.uk/PROSPERO/#, CRD42023415566.

## Introduction

Cancer, a condition characterized by a progressively escalating incidence of morbidity and mortality in recent years, particularly in the context of lung cancer, persists as the predominant global malignancy (1, 2). Within the realm of non-small cell lung cancer (NSCLC), the occurrence of metastasis to the central nervous system is noteworthy. The prevalence of brain metastases in initial NSCLC diagnoses ranges from 26 to 28%, escalating to affect 40–50% of patients during their ailment (3, 4). Evidently, this emergence significantly impacts prognostic outcomes and diminishes patients’ quality of life (3, 4). Indeed, the historical limitations imposed on therapeutic interventions for brain metastases stemmed from the formidable blood–brain barrier, characterized by the presence of tight junctions that effectively seal off the paracellular route between adjacent endothelial cells of cerebral capillaries (5). Notably, this barrier lacks the expression of active transport mechanisms, including pivotal efflux transporter proteins such as P-glycoprotein (P-gp), Breast Cancer Resistance Protein (BCRP), and organic anion-transporting peptides, which collectively regulate the passage of vital molecules, including essential amino acids induced by nutrient intake, while concurrently impeding the transit of undesired endogenous and exogenous substances (6). Furthermore, the existence of drug-metabolizing enzymes, particularly the cytochrome P450 (CYP450) enzymes within the cerebral endothelial cells, contributes to a substantial metabolic blockade (7), thereby constraining the feasibility of treating brain metastases through systemic pharmacotherapy alone. However, contemporary advancements in medical technology have substantially broadened the therapeutic armamentarium available for managing NSCLC brain metastases. This repertoire spans conventional approaches such as chemotherapy, surgical intervention, and WBRT, to state-of-the-art techniques including stereotactic radiotherapy (SRS), volumetric rotational radiotherapy, intensity-modulation technology, immunotherapy, and the highly anticipated targeted therapy. In particular, the advent of immunotherapy and targeted therapy represents the most recent inclusions in this expansive arsenal. Present research endeavors are firmly directed toward enhancing the efficacy of these treatments and augmenting patients’ overall survival rates.

Whole-brain radiotherapy and SRS constitute essential therapeutic modalities for patients suffering from brain metastases. The selection of the most appropriate radiotherapy regimen is primarily guided by various factors, including the size, location, number of brain lesions, and any concomitant neurological symptoms. Historically, since the 1950s, WBRT has prominently featured as the prevailing treatment for patients manifesting brain metastases (8). While conferring a noteworthy augmentation in intracranial lesion remission rates and amelioration of symptoms associated with intracranial hypertension, WBRT regrettably carries the burden of long-term cognitive impairment as an adverse consequence (9, 10). In the wake of technological advancements in SRS, certain scholarly circles have cast apprehension upon the appropriateness of WBRT utilization, a modality typically reserved for patients with limited, diminutive intracranial lesions. It has been posited that WBRT may potentially exert a safeguarding influence on cognitive function and enhance the overall quality of life for patients (9, 10). The investigations undertaken by the QUARTZ research group (11–13) have yielded insights indicating that, irrespective of the prognostic profile of patients afflicted with NSCLC brain metastases, the implementation of WBRT does not confer a survival advantage or improvements in quality of life compared to the application of best supportive care. Furthermore, WBRT has been associated with a heightened incidence of cognitive impairment in contrast to SRS. Consequently, there is an emerging suggestion that SRS may represent a more promising therapeutic avenue for this patient cohort. To mitigate the cognitive deficits associated with radiotherapy, numerous researchers have endeavored to develop contemporary techniques. Notably, analyses of two distinct studies, namely NRG CC001 and RTOG0614 (14, 15), have ascertained that patients undergoing hippocampal avoidance and receiving meglumine treatment exhibited an improved capacity for preserving cognitive function. Remarkably, this therapeutic approach did not exert a significant impact on intracranial progression-free survival or overall survival rates. Additionally, patients without metastatic involvement in the hippocampal region exhibited superior cognitive outcomes in this context, thereby warranting consideration as a potential standard of care.

The identification of biological targets has precipitated notable advancements in the management of patients afflicted with brain metastases. Preclinical investigations have unveiled that the anti-tumoral efficacy of radiotherapy can be enhanced through the incorporation of epidermal growth factor receptor tyrosine kinase inhibitors (EGFR-TKIs). These inhibitors possess the capacity to rectify the pathological vascularization of the tumor, mitigate tumor cell hypoxia, and heighten radiosensitivity. Consequently, the combination of radiotherapy and targeted agents engenders a synergistic effect on tumor suppression (16). Expanding on these preliminary findings, researchers worldwide have conducted phase II and III clinical trials. These trials aimed to assess the efficacy of EGFR-TKI in combination with WBRT, comparing it to WBRT alone in treating brain metastases originating from NSCLC. The outcomes have demonstrated that the combined therapeutic approach significantly attenuated intracranial lesions and yielded favorable intracranial responses, culminating in the extension of intracranial progression-free survival. Moreover, the management of short-term toxicity remained effectively controlled (17). Notwithstanding these promising results, a body of research has indicated the absence of substantial advantages in terms of both overall survival and progression-free survival (18, 19).

The efficacy and safety of the concomitant administration of EGFR-TKI and WBRT in contrast to WBRT as a monotherapy remain a matter of debate. While meta-analyses have been undertaken to investigate this issue, the outcomes lack conclusiveness due to the restriction in the scope of the literature considered and potential inaccuracies in the screening process. Consequently, there arises a necessity for an updated meta-analysis to facilitate a more precise assessment of the efficacy profile and toxicity response associated with the utilization of EGFR-TKI in conjunction with WBRT among individuals suffering from brain metastases stemming from NSCLC.

## Methods

This study was registered on PROSPERO (CRD42023415566). It adhered to the Preferred Reporting Items for Systematic Evaluations and Meta-Analysis (PRISMA) guidelines and complied with the recommendations set forth by the Cochrane Collaboration.

### Search strategy

A comprehensive systematic search of several prominent databases, including PubMed, Embase, Web of Science, Cochrane, Wanfang, and China National Knowledge Infrastructure (CNKI), was meticulously executed to identify clinical studies published until March 1, 2023, focusing on the utilization of EGFR-TKI in combination with WBRT for the management of brain metastases arising from NSCLC. The search strategy encompassed a wide array of pertinent search terms and keywords, including “Carcinoma,” “Non-Small Cell Lung,” “Non-Small-Cell Lung Carcinoma,” “Lung Carcinomas,” “Nonsmall Cell Lung Cancer,” “Brain Neoplasm,” “Brain Tumors,” “Brain Benign Neoplasm,” “Neoplasms, Brain, Benign,” “Neoplasms, Intracranial,” “Brain Tumor, Primary,” “Neoplasms, Brain, Primary,” “Brain Tumor, Recurrent,” “Malignant Primary Brain Tumors,” “Primary Malignant Brain Neoplasms,” “Brain Neoplasms, Malignant, Primary,” “Brain Metastases,” “Brain Cancer,” “Cancer, Brain,” “Malignant Neoplasms, Brain,” “Cancer of Brain,” “Radiotherapies,” “Radiation Therapy,” “Therapies, Radiation,” “Radiation Treatment,” “Treatment, Radiation,” “Radiotherapy, Targeted,” and “Targeted Radiation Therapy.” This comprehensive approach was adopted to ensure a thorough and exhaustive retrieval of relevant clinical studies in the specified domain. The detailed retrieval process is illustrated in Supplementary material 1.

### Eligibility criteria

#### Inclusion criteria

1. Study design: randomized controlled trials (RCTs) were included as the designated study design. The primary characteristic involved the random assignment of subjects into two distinct groups: one receiving the trial intervention of EGFR-TKI combined with WBRT, while the other group underwent WBRT alone as the control treatment. The principal objective was to compare the efficacy of the trial treatment against the control treatment.
2. Participants: patients diagnosed with brain metastases originating from NSCLC, as confirmed through both pathological and imaging methods.
3. Interventions: the experimental group received treatment comprising EGFR-TKIs in combination with WBRT, while the control group underwent WBRT as monotherapy.
4. The study should encompass multiple outcome measures, which may include the intracranial objective response rate (iORR) = complete response (CR) + partial response (PR), intracranial disease control rate (iDCR) = CR + PR + stable disease (SD), 1-year survival rate, and assessments of treatment-related toxicities. It is imperative that at least one of these measures be employed for an accurate evaluation of treatment efficacy and safety.

#### Exclusion criteria

1. Studies incorporating EGFR-TKI in conjunction with concurrent modalities, such as chemotherapy or immunotherapy.
2. Data originating from sources such as animal experiments, foundational research endeavors, or case reports, which may lack relevance to the subject under investigation.
3. Studies adopting a single-arm approach with the employment of solely one treatment arm.

### Data extraction

Each author contributed to the formulation of the literature search methodology. Two researchers, working independently, conducted the comprehensive review of identified literature. They jointly determined the studies that satisfied the predefined inclusion criteria and performed the data extraction. In instances where discordance arose, resolution was achieved through collaborative deliberation among team members. The extracted data encompassed information pertaining to the authors, publication year, study design, sample size, treatment modalities employed, and the various outcome measures evaluated. Any absent or unavailable data within the original literature sources were denoted as “NA” in the records.

### Study quality assessment

The two authors evaluated the literature quality of the included RCTs using the Cochrane Risk of Bias (ROB) tool. This tool assesses the following six key aspects: (1) selection bias, evaluating the random sequence generation and allocation concealment; (2) implementation bias, assessing the blinding of subjects and trial staff; (3) measurement bias, appraising the actual blinding of outcome assessors; (4) follow-up bias, considering incomplete outcome data; (5) reporting bias, examining selective reporting of study results; and (6) other factors potentially causing bias. Each section was categorized as exhibiting low, unclear, or high risk based on the respective article. Discordance between assessments was resolved through consensus reached via deliberation among the team members. The literature quality assessment is shown in Table 1.

**Table 1:** Study characteristics of the 23 eligible articles.

Author (year)	Study design	Patient		Intervene	Duration of treatment	outcome	EGFR mutation (T/C, %)	Risk of bias
		Treatment group	Control group					
Han (20)	RCT	37	36	125 mg Icotinib + WBRT (30Gy/10F) vs. WBRT (30Gy/10F)	4 weeks	iDCR, toxicity	100/100	Unclear
Shen (21)	RCT	36	36	150 mg Erlotinib + WBRT (40Gy/20F) vs. WBRT (40Gy/20F)	Disease progression or intolerable toxicity	iORR, iDCR, 1-Year survival rate, and toxicity	100/NA	Unclear
Ma et al. (22)	RCT	45	44	250 mg Gefitinib + WBRT (30Gy/10F) vs. WBRT (30Gy/10F)	Disease progression or intolerable toxicity	iORR, iDCR, and toxicity	NA/NA	High
Xi (23)	RCT	22	22	150 mg Erlotinib + WBRT (40Gy/20F) + Local addition (10Gy/5F) vs. WBRT (40Gy/20F) + Local addition (10Gy/5F)	4 weeks	iORR, iDCR, and toxicity	NA/NA	Unclear
Qi (24)	RCT	24	24	125 mg Icotinib + WBRT (NA) vs. WBRT (NA)	NA	iDCR, toxicity	NA/NA	Low
Xu (25)	RCT	31	31	250 mg Gefitinib + WBRT (30Gy/10F) vs. WBRT (30Gy/10F)	Disease progression or intolerable toxicity	iDCR, 1-Year survival rate, and toxicity	100/100	Low
Yang et al. (18)	RCT	106	114	150 mg Erlotinib + WBRT (40Gy/20F) vs. WBRT (40Gy/20F)	Disease progression or intolerable toxicity	Toxicity	54.7/44.7	Unclear
Xie (26)	RCT	37	37	250 mg Gefitinib + WBRT (NA) vs. WBRT (NA)	NA	iORR, iDCR, 1-Year survival rate, and toxicity	NA/NA	High
Huang et al. (27)	RCT	39	39	125 mg Icotinib + WBRT (30Gy/10F) vs. WBRT (30Gy/10F)	Disease progression or intolerable toxicity	Toxicity	NA/NA	High
Huang and Jiang (28)	RCT	250	250	150 mg Erlotinib + WBRT (30Gy/10F) vs. WBRT (30Gy/10F)	NA	iORR, iDCR, 1-Year survival rate, and toxicity	NA/NA	High
Ji et al. (29)	RCT	23	21	125 mg Icotinib + WBRT (30Gy/10F) + Local addition (5-12Gy) vs. WBRT (30Gy/10F) Local addition (5-12Gy)	Disease progression or intolerable toxicity	iORR, iDCR, and toxicity	56.5/42.9	Low
Xiao (30)	RCT	25	25	80 mg osimertinib + WBRT (30Gy/10F) vs. WBRT (30Gy/10F)	Disease progression or intolerable toxicity	iORR, iDCR, and toxicity	NA/NA	Unclear
Zheng (31)	RCT	22	22	250 mg Gefitinib + WBRT (30Gy/10F) vs. WBRT (30Gy/10F)	8 weeks	iORR, iDCR, and toxicity	100/100	High
Lu (32)	RCT	45	45	150 mg Erlotinib + WBRT (30Gy/10F) vs. WBRT (30Gy/10F)	2 weeks	iORR, iDCR, 1-Year survival rate, and toxicity	NA/NA	Unclear
Wang (33)	RCT	30	30	250 mg Gefitinib + WBRT (40Gy/20F) vs. WBRT (40Gy/20F)	4 weeks	iORR, iDCR, and toxicity	NA/NA	Unclear
Xue et al. (34)	RCT	31	30	250 mg Gefitinib + WBRT (30Gy/10F) vs. WBRT (30Gy/10F)	8 weeks	iORR, iDCR, and toxicity	100/100	Unclear
Zhang (35)	RCT	28	28	150 mg Erlotinib + WBRT (30Gy/10F) vs. WBRT (30Gy/10F)	2 weeks	iORR, iDCR, 1-Year survival rate, and toxicity	NA/NA	Unclear
Xu (36)	RCT	32	32	250 mg Gefitinib + WBRT (40Gy/20F) + Local addition (10Gy/5F) vs. WBRT (40Gy/20F) + Local addition (10Gy/5F)	12 weeks	iORR, iDCR, and toxicity	NA/NA	Unclear
Yuan and Hao (37)	RCT	23	23	150 mg Erlotinib + WBRT (40Gy/20F) + Local addition (10Gy/5Gy) vs. WBRT (40Gy/20F) + Local addition (10Gy/5Gy)	4 weeks	iORR, iDCR, toxicity, and 1-Year survival rate	NA/NA	Unclear
Liang et al. (38)	RCT	35	35	150 mg Erlotinib + WBRT (40Gy/20F) vs. WBRT (40Gy/20F)	Disease progression or intolerable toxicity	iDCR, toxicity	100/100	Low
Zhang (39)	RCT	20	20	250 mg Gefitinib + WBRT (40Gy/20F) vs. WBRT (40Gy/20F)	Disease progression or intolerable toxicity	iDCR, 1-Year survival rate, and toxicity	NA/NA	High
Zou and Tao (40)	RCT	30	30	250 mg Gefitinib + WBRT (40Gy/20F) vs. WBRT (40Gy/20F)	Disease progression or intolerable toxicity	iORR, iDCR, 1-Year survival rate, and toxicity	100/100	Unclear
Lee et al. (19)	RCT	40	40	100 mg Erlotinib + WBRT (20Gy/10F) vs. WBRT (20Gy/10F)	Disease progression or intolerable toxicity	Toxicity	NA/NA	Low

### Statistical analysis

The analysis of outcome indicators was conducted utilizing Stata 17.0 software. Dichotomous variables underwent scrutiny with the odds ratio (OR) serving as the analytical statistic. The inclusion of studies with substantial clinical heterogeneity due to variations in study protocols, baseline patient profiles, specific EGFR-TKI types, and divergent methods of split-dose whole-brain radiotherapy is noteworthy. Hence, despite the potential presence of statistical heterogeneity, we proceeded with the data analysis by employing the random-effects model. Additionally, we conducted subgroup analyses based on drug types to further investigate the origins of heterogeneity. To ensure the robustness and consistency of the meta-analysis findings, a sensitivity analysis was performed, systematically excluding each literature piece one by one. Any reversed articles were identified as potential sources of heterogeneity. Moreover, we employed Egger’s test to detect potential publication bias, revealing a significance level of *p* < 0.05. Consequently, we utilized an iterative approach to estimate the number of missing studies and address any underlying publication bias concerns.

## Results

### Study selection and characteristics

**Figure 1:**
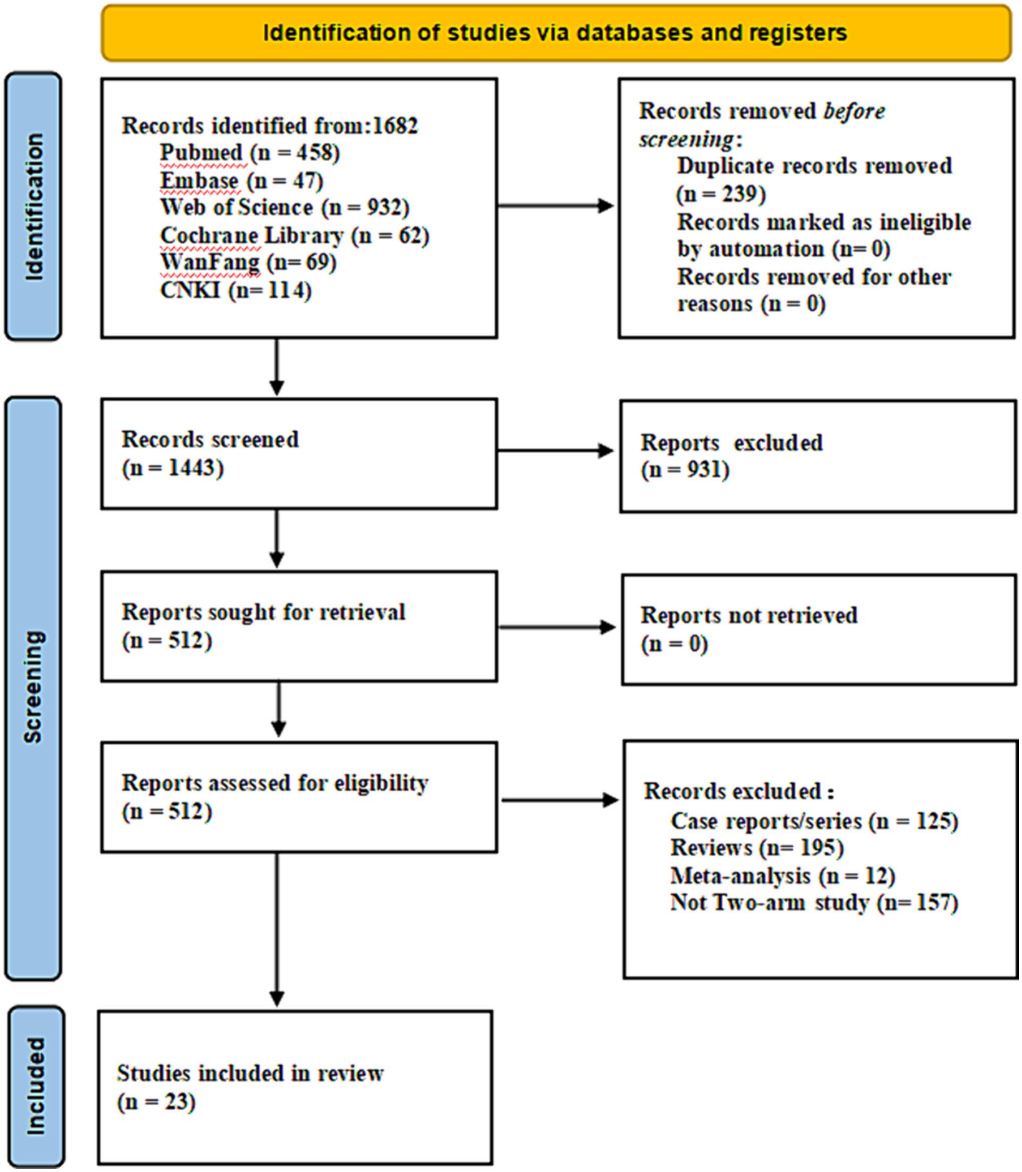
Flow chart of literature screening.

A comprehensive search strategy yielded a total of 1,682 documents, distributed among various databases: 458 from PubMed, 47 from Embase, 932 from Web of Science, 62 from the Cochrane library, 69 from Wanfang, and 114 from CNKI. After the removal of duplicate literature, 1,443 articles were retained. Subsequently, following the evaluation of titles and abstracts, these articles were excluded. Ultimately, 23 articles were included for review (Figure 1). All patients in the literature underwent whole-brain radiotherapy, with varying split-dose regimens: 11 papers (20, 22, 25, 27–32, 34, 35) had a split dose of 30Gy/10F, nine papers (18, 21, 23, 33, 36–40) employed 40Gy/20F, and one paper (19) used 20Gy/10F, while two papers (24, 26) did not specify the split dose. Additionally, three papers (23, 29, 36) targeted intracranial metastases with a localized add-on dose of 10Gy/5F.

In all literature, patients received EGFR-TKI treatment. Nine papers (22, 25, 26, 31, 33, 34, 36, 39, 40) administered oral 250 mg of gefitinib, with the continuation until disease progression or intolerable toxicity in four papers (22, 25, 39, 40). Two papers (31, 34) used it for 8 weeks, one (33) for 4 weeks, one (36) for 12 weeks, and one (26) did not specify the duration. Nine other papers (18, 19, 21, 23, 28, 32, 35, 37, 38) used 125 mg of erlotinib orally, except for one (19), of which four (18, 19, 21, 38) were administered until disease progression or intolerable toxicity, two (23, 37) were administered for 4 weeks, two (32, 35) were administered for 2 weeks, and one (28) was not elucidated for duration of administration; four papers (20, 24, 27, 29) used oral 125 mg icotinib, of which two papers (27, 29) used it until disease progression or intolerable toxicity, one paper (20) used it for 4 weeks, and one paper (24) did not elaborate on the duration of continued use. One paper (30) used oral 80 mg osimertinib until disease progression or intolerable toxicity. Seven studies (20, 21, 25, 31, 34, 38, 40) included patients with EGFR mutations, and 14 studies (19, 22–24, 26–28, 30, 32, 33, 35–37, 39) omitted the description of the EGFR status among patients. One article (18) reported EGFR rates, where the experimental and control groups displayed rates of 54.7 and 44.7%, respectively. Another article (29) noted EGFR rates with the experimental and control groups at 56.5 and 42.9%, respectively. The quality of the included literature was evaluated by the Cochrane ROB tool, where 12 studies (18, 20, 21, 23, 30, 32–37, 40) were classified as unclear, and six studies (22, 26–28, 31, 39) posed a high risk. Meanwhile, five articles (19, 24, 25, 29, 38) were deemed low risk. All studies contained extractable data on at least one of the following: iORR, iDCR, 1-year survival, and toxic and side effects. Table 1 presents the baseline characteristics of the included studies.

### Meta-analysis results

#### Intracranial objective remission rate

**Figure 2:**
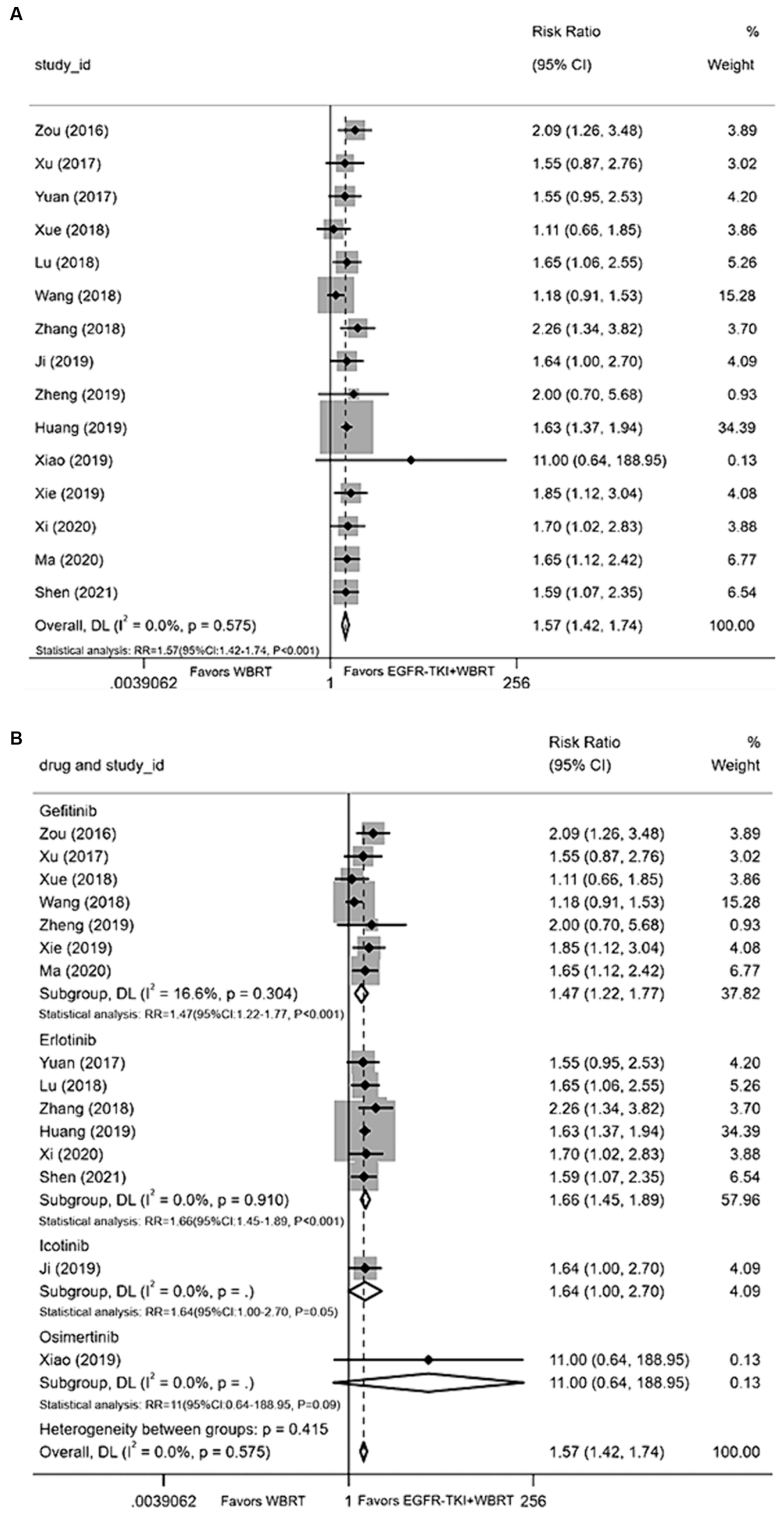
**(A)** Comparison of the intracranial objective response rate after treatment between the EGFR-TKI combined with WBRT group and the WBRT alone group. **(B)** Subgroup analysis categorized by different medications.

Fifteen articles (21–23, 26, 28–37, 40) provided data on the iORR. A random-effects model was applied to consolidate the statistics, revealing a statistically significant difference in iORR between EGFR-TKI in combination with WBRT and WBRT alone, with minimal heterogeneity (*I*^2^ = 0.0%, *p* = 0.575). The RR was 1.57 (95% CI: 1.42–1.74, *p* < 0.001; Figure 2A). Further analysis involved subgrouping various types of EGFR-TKI drugs. The results indicated notable effects for gefitinib (RR = 1.47, 95% CI: 1.22–1.77, *p* < 0.001; Figure 2B), erlotinib (RR = 1.66, 95% CI: 1.45–1.89, *p* < 0.001; Figure 2B), and icotinib (RR = 1.64, 95% CI: 1.00–2.7, *p* = 0.05). The combination of whole-brain radiotherapy and EGFR-TKIs (Figure 2B) exhibited a significantly improved iORR compared to WBRT alone. However, the osimertinib group did not manifest a significant advantage (RR = 11, 95% CI: 0.64–188.95, *p* = 0.09; Figure 2B).

#### Intracranial disease control rate

**Figure 3:**
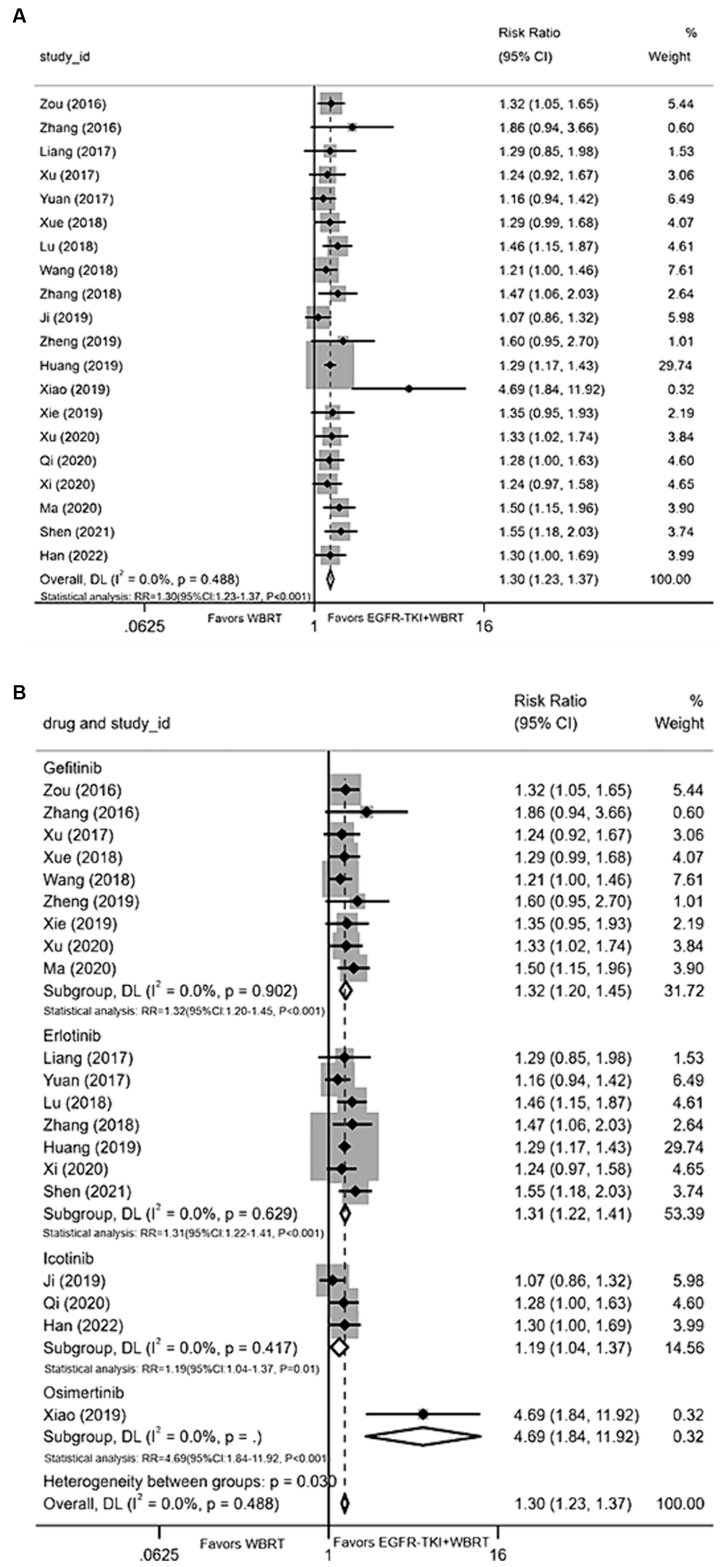
**(A)** Comparison of the intracranial disease control rate after treatment between the EGFR-TKI combined with WBRT group and the WBRT alone group. **(B)** Subgroup analysis categorized by different medications.

Twenty articles (20–26, 28–40) presented data on the iDCR. Employing a random-effects model for amalgamating the statistics, the analysis revealed negligible heterogeneity (*I*^**2**^ = 0.0%, *p* = 0.488). It was demonstrated that the intracranial control achieved with EGFR-TKI in conjunction with WBRT conferred a significant advantage compared to WBRT alone, yielding a RR of 1.30 (95% CI: 1.23–1.37, *p* < 0.001, Figure 3A). Subsequently, a subgroup analysis was conducted considering various types of EGFR-TKI drugs, with terminologies explained upon their initial use. The findings demonstrated superior iDCR when gefitinib (RR = 1.32, 95% CI: 1.20–1.45, *p* < 0.001; Figure 3B), erlotinib (RR = 1.31, 95% CI: 1.22–1.41, *p* < 0.001; Figure 3B), icotinib (RR = 1.19, 95% CI: 1.04–1.37, *p* = 0.01; Figure 3B), and osimertinib were combined with WBRT in comparison to WBRT alone in terms of iDCR (RR = 4.69, 95% CI: 1.84–11.92, *p* < 0.001; Figure 3B).

#### 1-year survival rate

**Figure 4:**
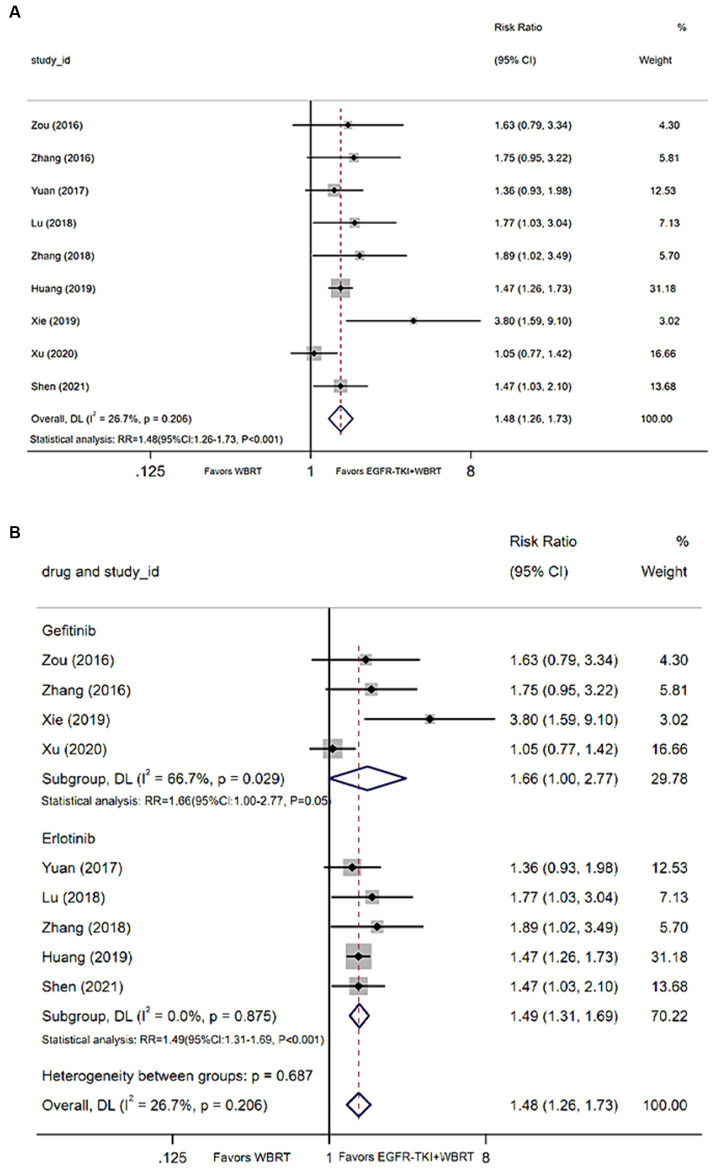
**(A)** Comparison of 1-year survival rate after treatment between the EGFR-TKI combined with WBRT group and the WBRT alone group. **(B)** Subgroup analysis categorized by different medications.

Nine articles (21, 25, 26, 28, 32, 35, 37, 39, 40) contributed data on the 1-year survival rate. Upon conducting a meta-analysis with a random-effects model, the findings indicated a statistically significant enhancement in 1-year survival rates when employing EGFR-TKI in conjunction with WBRT, as opposed to WBRT alone. The analysis yielded a modest degree of heterogeneity (*I*^2^ = 26.7%, *p* = 0.206). The RR was 1.48 (95% CI: 1.26–1.73, *p* < 0.001, Figure 4A). For further analysis, different types of EGFR-TKI drugs were grouped for further investigation. The findings suggested that the 1-year survival rate was notably higher when using combined erlotinib (RR = 1.49, 95% CI: 1.31–1.69, *p* < 0.001; Figure 4B) compared to gefitinib (RR = 1.66, 95% CI: 1.00–2.77, *p* = 0.05; Figure 4B) in conjunction with WBRT, as opposed to WBRT alone.

#### Incidence of adverse reactions

**Figure 5:**
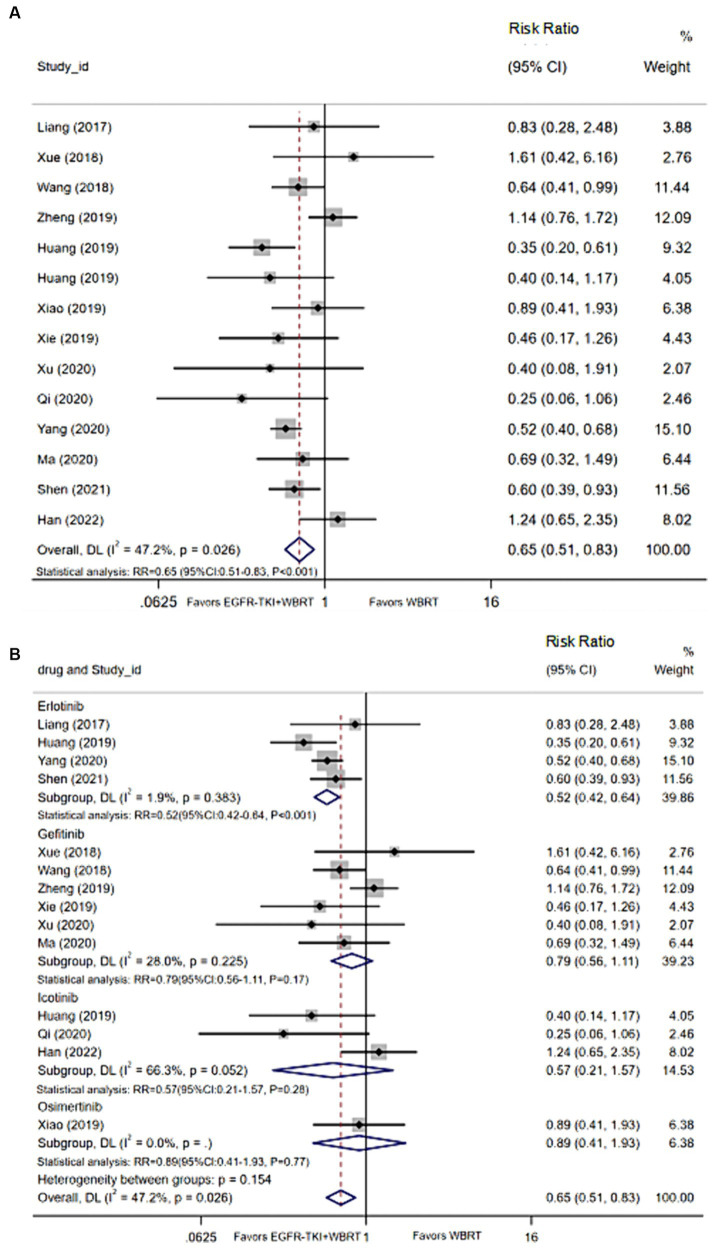
**(A)** Comparison of the incidence of adverse reactions after treatment between the EGFR-TKI combined with WBRT group and the WBRT alone group. **(B)** Subgroup analysis categorized by different medications.

Twenty-three articles (18–40) provided data on toxic reactions, whereas 14 articles (18, 20–22, 24–28, 30, 31, 33, 34, 38) reported the incidence of adverse reactions. Utilizing a random-effects model to amalgamate the statistics, we observed a moderate degree of heterogeneity (*I*^2^ = 47.2%, *p* = 0.026). The analysis indicated that the incidence of adverse reactions with EGFR-TKI in combination with WBRT, as compared to WBRT alone, did exhibit statistically significant differences (RR = 0.65, 95% CI: 0.51–0.83, *p* < 0.001; Figure 5A). Further exploration included subgroup analysis based on different EGFR-TKI drug types. The outcomes highlighted that, in comparison to WBRT alone, the combination with gefitinib (RR = 0.79, 95% CI: 0.56–1.11, *p =* 0.17; Figure 5B), osimertinib (RR = 0.89, 95% CI: 0.41–1.93, *p* = 0.77; Figure 5B), icotinib (RR = 0.57, 95% CI: 0.21–1.57, *p* = 0.28; Figure 5B), or erlotinib (RR = 0.52, 95% CI: 0.42–0.64, *p* < 0.001; Figure 5B), especially in combination with erlotinib, reduced adverse events.

### Common adverse events observed

#### Myelosuppression

**Figure 6:**
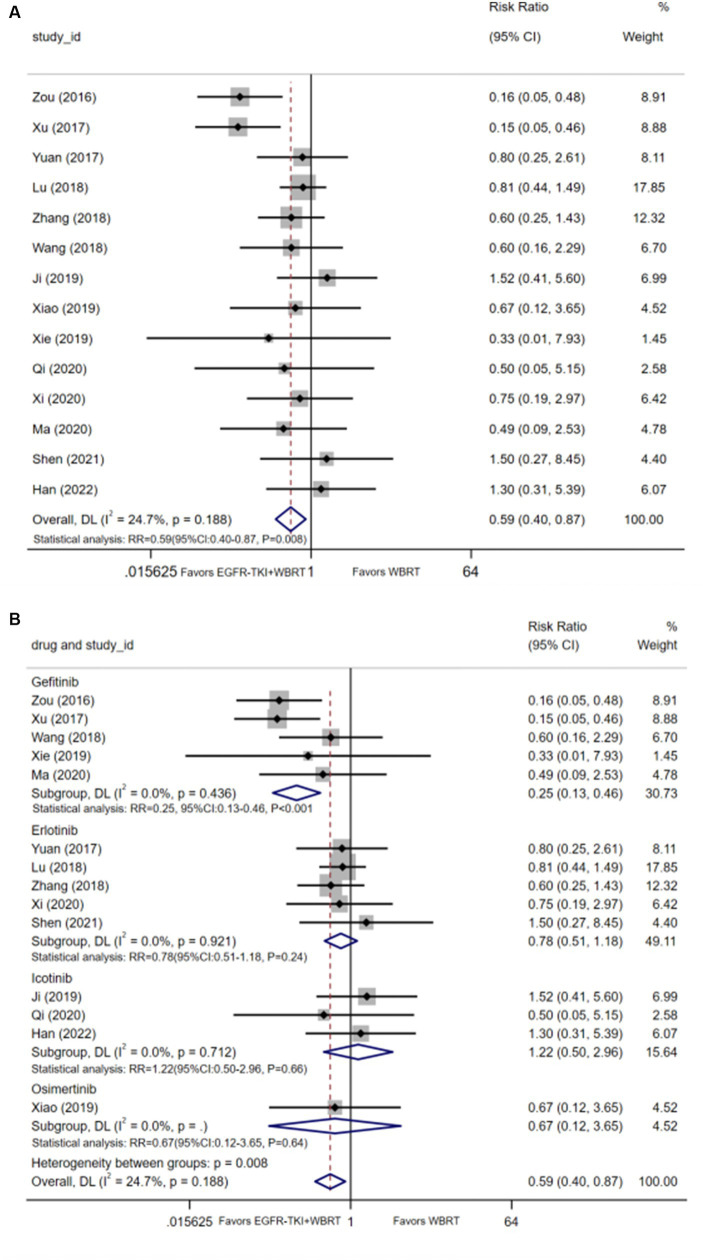
**(A)** Comparison of incidence of myelosuppression after treatment between the EGFR-TKI combined with WBRT group and the WBRT alone group. **(B)** Subgroup analysis categorized by different medications.

14 articles (20–24, 26, 29, 30, 32, 33, 35–37, 40) detailed the incidence of myelosuppression, and the synthesis of statistics using a random-effects model displayed negligible heterogeneity (*I*^2^ = 24.7%, *p* = 0.188). The analysis demonstrated statistically significant differences in the incidence of myelosuppression between EGFR-TKI combined with WBRT and WBRT alone (RR = 0.59, 95% Cl: 0.40–0.87, *p* = 0.008, Figure 6A). Subsequent subgroup analysis based on various types of EGFR-TKI drugs revealed comparable incidences of myelosuppression when comparing the combination therapies to WBRT alone. Further analysis involved subgrouping various types of EGFR-TKI drugs for a detailed examination. The results revealed a statistically significant discrepancy in the incidence of myelosuppression when combined with gefitinib (RR = 0.25, 95% CI: 0.13–0.46, *p* < 0.001, Figure 6B) compared to the administration of WBRT alone. However, no statistically significant differences were observed in the incidence of myelosuppression for icotinib (RR = 1.22, 95% CI: 0.50–2.96, *p* = 0.66, Figure 6B), erlotinib (RR = 0.78, 95% CI: 0.51–1.18, *p* = 0.24, Figure 6B), and osimertinib (RR = 0.67, 95% CI: 0.12–3.65, *p* = 0.64, Figure 6B).

#### Nausea and vomiting

**Figure 7:**
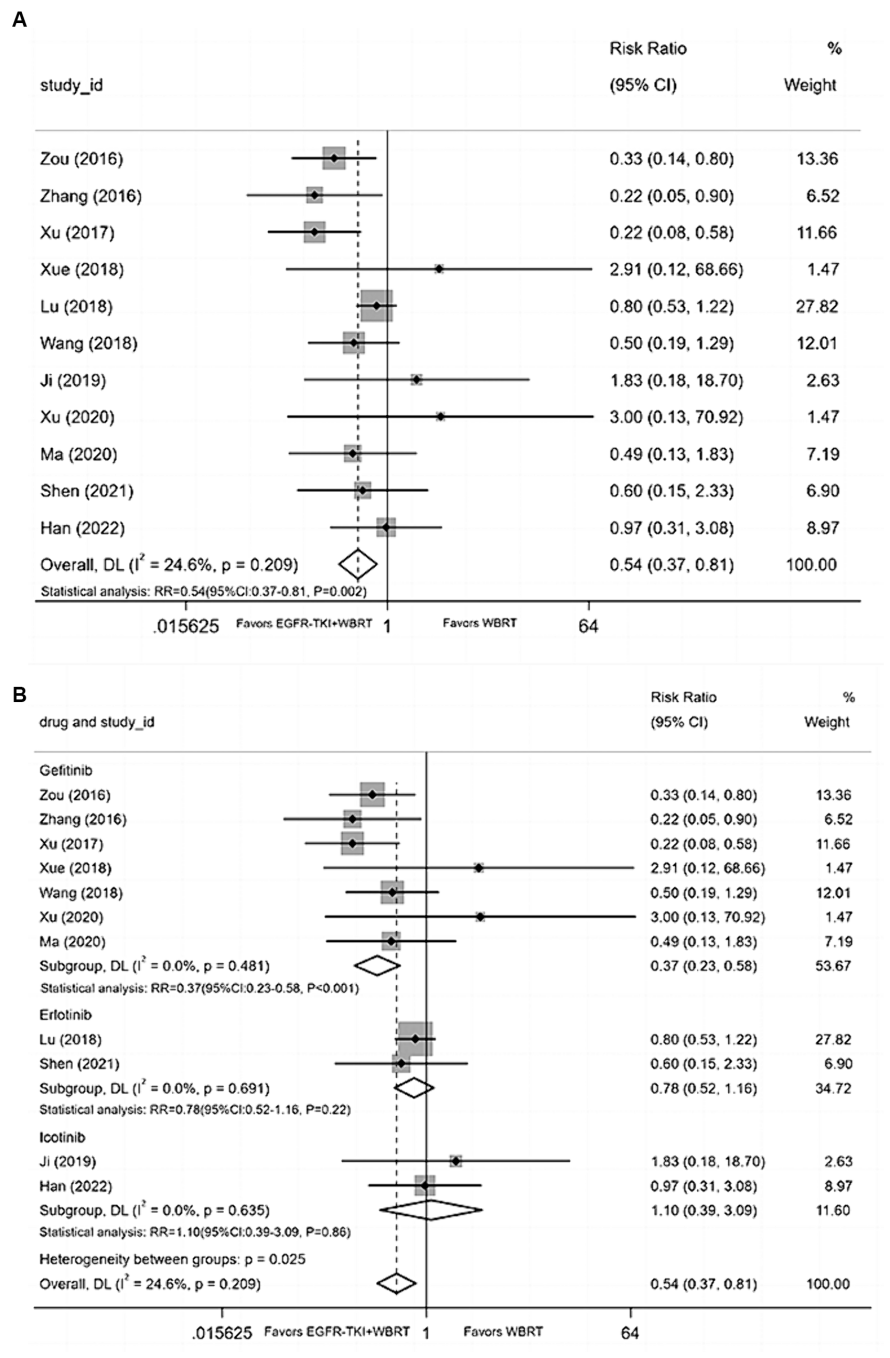
**(A)** Comparison of the incidence of nausea and vomiting after treatment between the EGFR-TKI combined with WBRT group and the WBRT alone group. **(B)** Subgroup analysis categorized by different medications.

Eleven articles (20–22, 25, 29, 32–34, 36, 39, 40) reported data on the incidence of nausea and vomiting, with a heterogeneity level of *I*^2^ = 24.6% and *p* = 0.209. The analysis revealed a statistically significant difference in the incidence of nausea and vomiting between EGFR-TKI combined with WBRT and WBRT alone (RR = 0.54, 95% CI: 0.37–0.81, *p* = 0.002; Figure 7A). Subsequent subgroup analysis based on distinct types of EGFR-TKI drugs signified a notable reduction in the incidence of nausea and vomiting when combined with gefitinib (RR = 0.37, 95% CI: 0.23–0.58, *p* < 0.001; Figure 7B) compared to WBRT alone. However, statistical differences were not observed in the incidence of nausea and vomiting for icotinib (RR = 1.10, 95% CI: 0.39–3.09, *p* = 0.86; Figure 7B) and erlotinib (RR = 0.78, 95% CI: 0.52–1.16, *p* = 0.22; Figure 7B) when combined with WBRT.

#### Diarrhea

**Figure 8:**
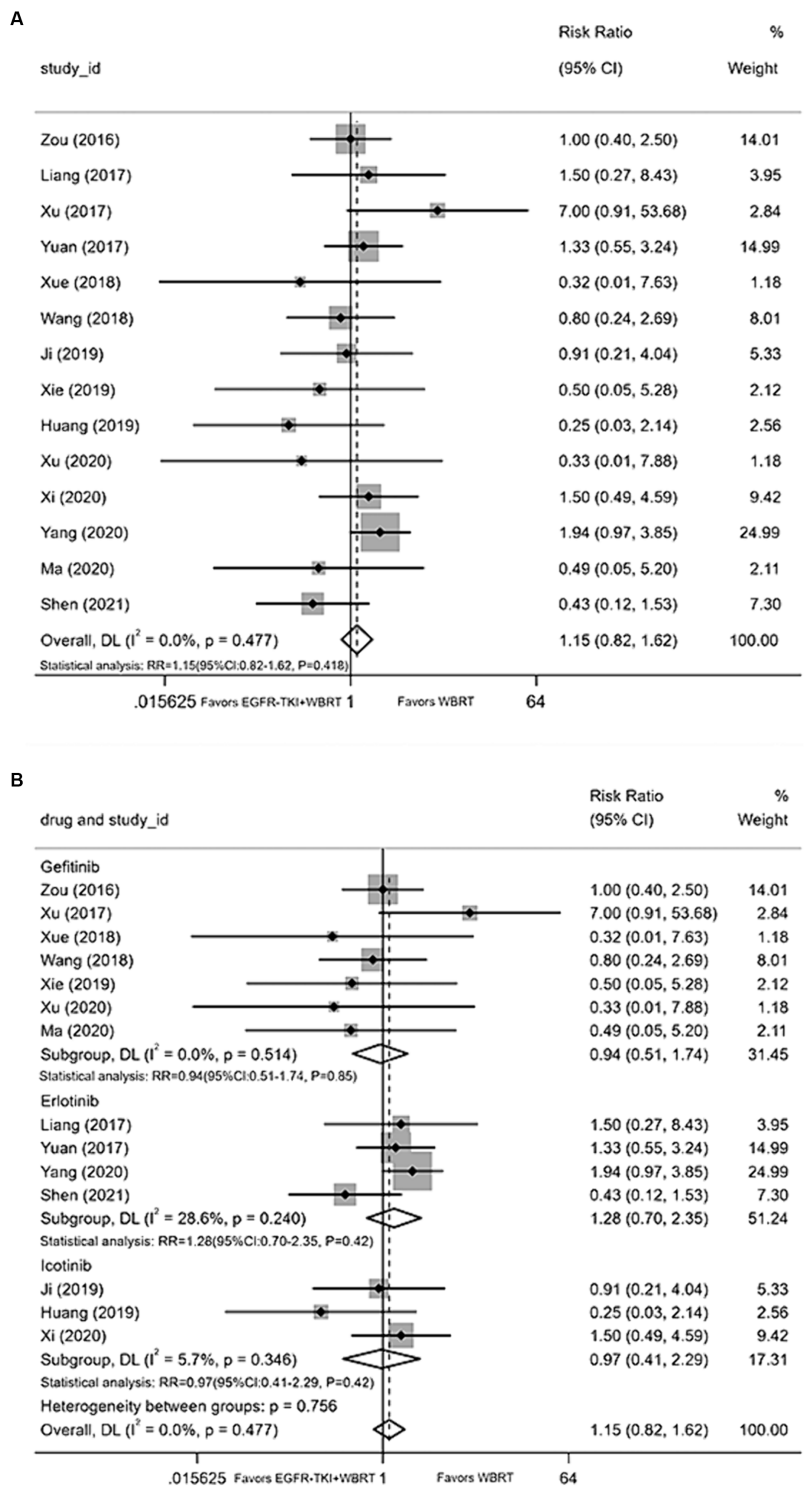
**(A)** Comparison of incidence of diarrhea after treatment between the EGFR-TKI combined with WBRT group and the WBRT alone group. **(B)** Subgroup analysis categorized by different medications.

Fourteen articles (18, 21–23, 25–27, 29, 33, 34, 36–38, 40) contributed data on the incidence of diarrhea, employing a random-effects model to merge statistics. The analysis indicated that the incidence of diarrhea between EGFR-TKI combined with WBRT and WBRT alone did not demonstrate statistically significant differences, exhibiting minimal heterogeneity (*I*^2^ = 0.0%, *p* = 0.477; RR = 1.15, 95% CI: 0.82–1.62, *p* = 0.418; Figure 8A). Subgroup analysis was conducted by grouping different types of EGFR-TKI drugs for further investigation. The findings revealed no statistically significant differences in the incidence of diarrhea when combined with gefitinib (RR = 0.94, 95% CI: 0.51–1.74, *p* = 0.85; Figure 8B), icotinib (RR = 0.97, 95% CI: 0.41–2.29, *p* = 0.42; Figure 8B), or erlotinib (RR = 1.28, 95% CI: 0.70–2.35, *p* = 0.42; Figure 8B) compared to WBRT alone.

#### Rash

**Figure 9:**
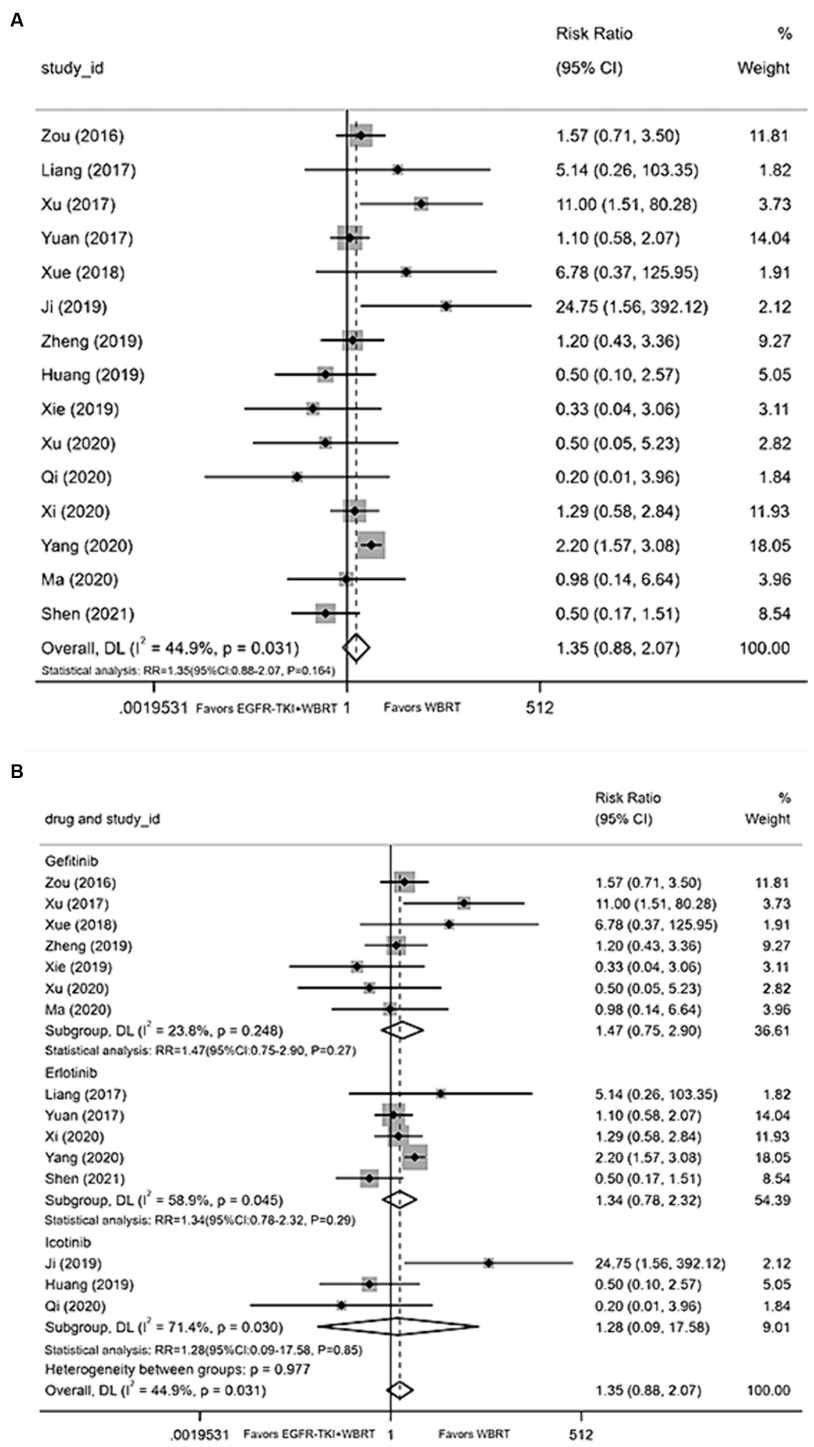
**(A)** Comparison of the incidence of skin rash after treatment between the EGFR-TKI combined with WBRT group and the WBRT alone group. **(B)** Subgroup analysis categorized by different medications.

Fifteen articles (18, 21–27, 29, 31, 34, 36–38, 40) furnished data on the incidence of rash. The application of a random-effects model for statistical consolidation indicated a moderate level of heterogeneity (*I*^**2**^ = 44.9%, *p* = 0.031). The results revealed no statistically significant disparities in the incidence of rash between EGFR-TKI in combination with WBRT and WBRT alone (RR = 1.35, 95% CI: 0.88–2.07, *p* = 0.164; Figure 9A). Further analysis involved grouping various EGFR-TKI drugs for subgroup analysis. The study found no statistically significant difference in the incidence of rash when combined with gefitinib (RR = 1.47, 95% CI: 0.75–2.90, *p* = 0.27; Figure 9B), icotinib (RR = 1.28, 95% CI: 0.09–17.58, *p* = 0.85; Figure 9B), or erlotinib (RR = 1.34, 95% CI: 0.78–2.32, *p* = 0.29; Figure 9B) compared to WBRT alone.

### Sensitivity analysis and publication bias

Sequential exclusion of one document resulted in the aggregation of the remaining documents (n−1) for meta-analysis. Results from sensitivity analyses demonstrated that no individual study significantly influenced the outcomes, indicating their stability (Supplementary material 2).

The iDCR served as the outcome measure, and Egger’s test identified a publication bias (*p* = 0.021). Employing an iterative method, an estimation suggested an absence of six studies. Consequently, data from six dummy studies were incorporated, encompassing all studies in the meta-analysis. However, the findings remained consistent (RR = 1.27, 95% CI: 1.20–1.33, *p* < 0.001), underscoring the robustness of the outcomes. Notably, no publication bias was detected in the assessment of other outcome indicators.

## Discussion

Cranial radiotherapy continues to serve as the established therapeutic modality for patients afflicted with brain metastases originating from NSCLC. WBRT or SRS effectively mitigates the distressing manifestations of intracranial hypertension, such as nausea, vomiting, dizziness, and headaches. Regrettably, the presence of brain metastases signifies a dismal prognosis, characterized by a truncated survival duration, typically spanning a median of approximately 4–7 months (41). In recent years, researchers have persistently engaged in clinical trials assessing the confluence of WBRT with conventional chemotherapeutic agents like temozolomide and pemetrexed. The outcomes of these trials have not yielded a significant enhancement in overall patient survival. This lack of substantial benefit can predominantly be ascribed to the limited ability of conventional chemotherapeutic agents to traverse the blood–brain barrier (42–45). Contrarily, tyrosine kinase inhibitors such as erlotinib, gefitinib, and icotinib possess a notable degree of permeability through the blood–brain barrier. Moreover, they exhibit the property of radiosensitization, thereby manifesting anti-tumoral effects (16, 46). The combination of EGFR-TKIs with WBRT appears to yield superior outcomes in patients afflicted with brain metastases originating from NSCLC. Consequently, this amalgamated approach furnishes a promising therapeutic avenue for these patients.

The utilization of EGFR-TKIs in combination with WBRT among patients afflicted with brain metastases stemming from NSCLC holds the potential to elicit an intracranial response. However, the presence of diverse treatment modalities across clinical trials can engender a convergence of disparate findings, leading to divergent conclusions. Our study affirms that the incorporation of EGFR-TKIs alongside WBRT confers a potential advantage, characterized by enhanced objective intracranial responses and favorable side effect profiles. Our meta-analysis, encompassing 23 included articles, demonstrates that EGFR-TKI combined with WBRT yields superior outcomes in terms of iORR (RR = 1.57, 95% CI: 1.42–1.74, *p* < 0.001) and iDCR (RR = 1.30, 95% CI: 1.23–1.37, *p* < 0.001) when juxtaposed with WBRT alone. Furthermore, we observe a significant enhancement in 1-year survival rate (RR = 1.48, 95% CI: 1.26–1.73, *p* < 0.001) upon the addition of EGFR-TKIs, with tolerable adverse events (RR = 0.65, 95% CI: 0.51–0.83, *p* < 0.001). We conducted an exhaustive analysis of the typical side effects and found that the incorporation of EGFR-TKI did not significantly increase the incidence of myelosuppression toxicity (RR = 0.59, 95% CI: 0.40–0.87, *p* = 0.008) or nausea and vomiting (RR = 0.54, 95% CI: 0.37–0.81, *p* = 0.002). Additionally, there were no statistically significant differences observed in other prevalent adverse effects between both cohorts. In this study, we sequentially excluded one literature and merged the remaining studies (n−1) through meta-analysis. The sensitivity analysis demonstrated that no individual study significantly influenced the outcomes, underscoring the robustness and stability of our final conclusions derived from the meta-analysis. Furthermore, we conducted subgroup analysis by categorizing various types of EGFR-TKI drugs. Our findings indicate that combining different EGFR-TKI drugs with WBRT exhibited a promising intracranial response with a low incidence of adverse events. Moreover, the incidence of common adverse events such as diarrhea and rash did not differ statistically, regardless of the use of gefitinib, erlotinib, or icotinib. Nevertheless, the incidence of nausea, vomiting, and myelosuppression was lower when administering gefitinib in combination with WBRT compared to sole WBRT. In addition, The minimal heterogeneity observed in this study might be associated with the quality of included studies, the split dosage of WBRT (Utilizing the differing radiation sensitivity and repair capabilities of tumor and normal cells, conventional radiotherapy employs fractionation, dividing a single large dose into smaller doses administered over time to optimize treatment outcomes), and other related factors. In particular, our study underscores the potential of combining EGFR-TKI with WBRT to mitigate adverse events in NSCLC patients with brain metastases. This observed reduction in adverse events can be attributed to several factors. First, radiation primarily works by inducing a response that damages the DNA, suppressing tumor cell proliferation, and promoting cell apoptosis (47). However, the radiation-induced DNA damage response may also trigger the release of cytokines, leading to an inflammatory reaction and further damaging surrounding tissues (48). Studies have shown that EGFR-TKIs may inhibit the activation of inflammatory cells and the release of cytokines, thereby mitigating the inflammatory response and cellular injury (49). Additionally, EGFR-TKIs can enhance the release and activation of vascular endothelial growth factor, promoting angiogenesis (50), which may aid in the regeneration and repair of radiation-induced tissue damage (51). Finally, the supplementation of EGFR-TKIs can enhance the therapeutic efficacy of radiotherapy by increasing the sensitivity of tumor cells to the cytotoxic effects (52), suggesting that combination therapy could potentially lower radiation doses and reduce radiation-related adverse reactions. Although our research results contradict traditional viewpoints, they indicate a favorable trend. Perhaps, in future studies, through further experimentation and increased sample sizes, we can obtain results that are more representative of real-world scenarios. Currently, ongoing investigations into mechanisms of EGFR mutation resistance reveal a potentially favorable safety profile for patients with brain metastases from NSCLC treated with third-generation EGFR-TKIs. However, disparities in efficacy levels and common adverse effects warrant further in-depth discussion.

The three generations of EGFR-TKI medications exhibit varied permeability across the blood–brain barrier, resulting in potential differences in their therapeutic efficacy. Tan et al. (53) conducted investigations into the efficacy of first-generation EGFR-TKIs, namely gefitinib, erlotinib, and icotinib, within intracranial transplant tumors. Their findings revealed that gefitinib exhibited a higher brain tumor-to-plasma concentration ratio in comparison to eclotinib and erlotinib. Consequently, gefitinib displayed superior proficiency in penetrating the blood-tumor barrier and disseminating within brain metastases. Subsequent to these theoretical underpinnings, two pivotal clinical trials, ICOGEN and CTONG0901, were conducted to assess the performance of first-generation EGFR-TKI agents, including gefitinib, icotinib, and erlotinib. These trials demonstrated some enhancements in both intracranial response and overall survival, yet the disparities observed did not reach statistical significance (54, 55). In the context of the second-generation EGFR-TKI, afatinib, the LUX-Lung7 trial failed to establish a statistically significant difference between gefitinib and afatinib in EGFR-positive patients afflicted with brain metastases, thus confirming the absence of a discernible intracranial therapeutic advantage with afatinib (56). The emergence of the third-generation EGFR-TKI, osimertinib, introduced a notable breakthrough, characterized by its heightened brain concentrations. Osimertinib exhibited a propensity to accumulate within the brain, attaining concentrations as high as 2.78 μmol/L, and demonstrated the capacity to effectively traverse the intact blood–brain barrier (57). Another clinical trial investigation underscored the substantial prolongation of the time to intracranial progression when osimertinib was employed in conjunction with intracranial radiotherapy (56). In summary, evidence substantiates that both initial and subsequent generations of EGFR-TKIs exhibit some penetration through the blood–brain barrier. However, the third generation demonstrates increased penetration and efficacy within the brain. The anticipation is that when combined with WBRT, this could demonstrate effective and sustained control over intracranial lesions, leading to an improved prognosis for patients and a reduced risk of mortality from the disease. Henceforth, it is strongly recommended that rigorous clinical designs incorporating prospective, randomized controlled trials for third-generation EGFR-TKIs (osimertinib, almonertinib, or furmonertinib) in combination with WBRT for patients with brain metastases from NSCLC. This will further validate their safety and efficacy profiles and offer an essential framework for clinicians in selecting EGFR-TKIs.

Undoubtedly, our meta-analysis bears certain limitations. Firstly, some of the studies we included did not specify the EGFR mutation status of patients. Therefore, it is possible that there are patients with EGFR-negative or unknown status who may be treated with EGFR-Tkis, but the efficacy of such patients is unclear. Secondly, despite the low heterogeneity observed in this study, the underlying causes were not further examined through subgroup analysis. This variance could potentially stem from several factors, including the year of publication, the quality of the studies included, variations in the type and duration of EGFR-TKI treatment, diverse WBRT dosages, EGFR mutation rates, and discrepancies in sample sizes across studies. Thirdly, publication bias remains a challenge to circumvent, particularly due to potential factors such as the omission of studies within the included population and small sample sizes. However, it is important to note that this does not substantially affect the conclusions drawn. Finally, most of the statistical data included in our study are mainly applicable to China, so there are certain regional limitations. To assess the extent of publication bias, we employed Egger’s test and utilized an iterative approach to estimate the number of potentially missing studies. In our analysis, we incorporated data from virtual studies, yet the overall findings remained consistent, indicating the robustness of the combined results. Consequently, it is imperative to integrate more potent and comprehensive evidence-based clinical reasoning in forthcoming analyses. Such an approach would enable medical practitioners to better evaluate the efficacy and safety of various therapeutic alternatives, establishing a foundation for optimal treatment strategies.

## Conclusion

This study demonstrated that EGFR-TKI combined with WBRT resulted in favorable intracranial responses, notably enhancing the objective intracranial response rate, disease control rate, and 1-year survival rate in comparison to WBRT alone among NSCLC patients with brain metastases. Furthermore, the incidence of adverse effects was not significantly higher, except for mild occurrences of rash and diarrhea. Based on these findings, it is recommended that employing third-generation EGFR-TKI in combination with WBRT stands as a preferable approach for patients suffering from brain metastases caused by NSCLC, facilitating optimal control over such metastases.

## Data availability statement

The original contributions presented in the study are included in the article/Supplementary material; further inquiries can be directed to the corresponding author.

## Author contributions

SL: Writing – original draft, Writing – review & editing. SX: Data curation, Methodology, Writing – review & editing. LL: Data curation, Methodology, Writing – review & editing. ZX: Data curation, Investigation, Writing – review & editing. LH: Data curation, Methodology, Writing – review & editing.
