# Supplementary material for: Efficacy and safety of EGFR-TKI combined with WBRT vs. WBRT alone in the treatment of brain metastases from NSCLC: a systematic review and meta-analysis
Source: Front Neurol. 2024 Apr 26;15:1362061. doi: 10.3389/fneur.2024.1362061 (PMC11085739; doi:10.3389/fneur.2024.1362061)
Supplement: Supplementary file 1 [file Data_Sheet_1.doc]

Supplementary materials 1 Search strategy
Pubmed 
#1  Search: "Carcinoma, Non-Small-Cell Lung"[Mesh] Sort by: Most Recent
#2  Search: ((((((((((Carcinoma, Non Small Cell Lung[Title/Abstract]) OR (Carcinomas, Non-Small-Cell Lung[Title/Abstract])) OR (Lung Carcinoma, Non-Small-Cell[Title/Abstract])) OR (Lung Carcinomas, Non-Small-Cell[Title/Abstract])) OR (Non-Small-Cell Lung Carcinomas[Title/Abstract])) OR (Non-Small-Cell Lung Carcinoma[Title/Abstract])) OR (Non Small Cell Lung Carcinoma[Title/Abstract])) OR (Carcinoma, Non-Small Cell Lung[Title/Abstract])) OR (Non-Small Cell Lung Carcinoma[Title/Abstract])) OR (Non-Small Cell Lung Cancer[Title/Abstract])) OR (Nonsmall Cell Lung Cancer[Title/Abstract])
#3   #1 OR #2
#4   Search: "Radiotherapy"[Mesh] Sort by: Most Recent
#5 Search: (((((((((((((((((Radiotherapies[Title/Abstract]) OR (Radiation Therapy[Title/Abstract])) OR (Radiation Therapies[Title/Abstract])) OR (Therapies, Radiation[Title/Abstract])) OR (Therapy, Radiation[Title/Abstract])) OR (Radiation Treatment[Title/Abstract])) OR (Radiation Treatments[Title/Abstract])) OR (Treatment, Radiation[Title/Abstract])) OR (Radiotherapy, Targeted[Title/Abstract])) OR (Radiotherapies, Targeted[Title/Abstract])) OR (Targeted Radiotherapies[Title/Abstract])) OR (Targeted Radiotherapy[Title/Abstract])) OR (Targeted Radiation Therapy[Title/Abstract])) OR (Radiation Therapies, Targeted[Title/Abstract])) OR (Targeted Radiation Therapies[Title/Abstract])) OR (Therapies, Targeted Radiation[Title/Abstract])) OR (Therapy, Targeted Radiation[Title/Abstract])) OR (Radiation Therapy, Targeted[Title/Abstract])
#6   #4 OR #5
#7   Search: "Brain Neoplasms"[Mesh] Sort by: Most Recent
#8 Search:((((((((((((((((((((((((((((((((((((((((((((((((((((Brain Neoplasm[Title/Abstract]) OR (Neoplasm, Brain[Title/Abstract])) OR (Neoplasms, Brain[Title/Abstract])) OR (Brain Tumors[Title/Abstract])) OR (Brain Tumor[Title/Abstract])) OR (Tumor, Brain[Title/Abstract])) OR (Benign Neoplasms, Brain[Title/Abstract])) OR (Benign Neoplasm, Brain[Title/Abstract])) OR (Brain Benign Neoplasm[Title/Abstract])) OR (Brain Benign Neoplasms[Title/Abstract])) OR (Neoplasms, Brain, Benign[Title/Abstract])) OR (Brain Neoplasms, Benign[Title/Abstract])) OR (Benign Brain Neoplasm[Title/Abstract])) OR (Benign Brain Neoplasms[Title/Abstract])) OR (Brain Neoplasm, Benign[Title/Abstract])) OR (Neoplasms, Intracranial[Title/Abstract])) OR (Intracranial Neoplasm[Title/Abstract])) OR (Neoplasm, Intracranial [Title/Abstract])) OR (Intracranial Neoplasms[Title/Abstract])) OR (Brain Tumor, Primary[Title/Abstract])) OR (Primary Brain Tumor[Title/Abstract])) OR (Primary Brain Tumors[Title/Abstract])) OR (Neoplasms, Brain, Primary[Title/Abstract])) OR (Brain Neoplasm, Primary[Title/Abstract])) OR (Primary Brain Neoplasms[Title/Abstract])) OR (Brain Neoplasms, Primary[Title/Abstract])) OR (Primary Brain Neoplasm[Title/Abstract])) OR (Brain Tumor, Recurrent[Title/Abstract])) OR (Brain Tumors, Recurrent[Title/Abstract])) OR (Recurrent Brain Tumor[Title/Abstract])) OR (Recurrent Brain Tumors[Title/Abstract])) OR (Malignant Primary Brain Tumors[Title/Abstract])) OR (Primary Malignant Brain Tumors[Title/Abstract])) OR (Malignant Primary Brain Neoplasms[Title/Abstract])) OR (Primary Malignant Brain Neoplasms[Title/Abstract])) OR (Brain Neoplasms, Malignant, Primary[Title/Abstract])) OR (Brain Neoplasms, Primary Malignant[Title/Abstract])) OR (Brain Metastases[Title/Abstract])) OR (Brain Metastase[Title/Abstract])) OR (Brain Cancer[Title/Abstract])) OR (Brain Cancers[Title/Abstract])) OR (Cancer, Brain[Title/Abstract])) OR (Malignant Neoplasms, Brain[Title/Abstract])) OR (Brain Malignant Neoplasm[Title/Abstract])) OR (Brain Malignant Neoplasms[Title/Abstract])) OR (Malignant Neoplasm, Brain[Title/Abstract])) OR (Cancer of Brain[Title/Abstract])) OR (Cancer of the Brain[Title/Abstract])) OR (Neoplasms, Brain, Malignant[Title/Abstract])) OR (Brain Neoplasms, Malignant[Title/Abstract])) OR (Brain Neoplasm, Malignant[Title/Abstract])) OR (Malignant Brain Neoplasm[Title/Abstract])) OR (Malignant Brain Neoplasms[Title/Abstract])
#9   #7 OR #8
#10  #3 AND #6 AND #9
#11 Search: prognosis[MeSH:noexp] OR diagnosed[Title/Abstract] OR cohort*[Title/Abstract] OR cohort[MeSH Terms] OR cohort studies[MeSH:noexp] OR predictor*[Title/Abstract] OR death[Title/Abstract] OR models, statistical[MeSH Term]
#12 Search: randomized controlled trial[Publication Type] OR randomized[Title/Abstract] OR placebo[Title/Abstract]
#13   #11 OR #12 AND #10

Web of science 
#1  (((((((((((TS=(Carcinoma, Non-Small-Cell Lung)) OR TS=(Carcinoma, Non Small Cell Lung)) OR TS=(Carcinomas, Non-Small-Cell Lung)) OR TS=(Lung Carcinoma, Non-Small-Cell)) OR TS=(Lung Carcinomas, Non-Small-Cell)) OR TS=(Non-Small-Cell Lung Carcinomas)) OR TS=(Non-Small-Cell Lung Carcinoma )) OR TS=(Non Small Cell Lung Carcinoma)) OR TS=(Carcinoma, Non-Small Cell Lung )) OR TS=(Non-Small Cell Lung Carcinoma )) OR TS=(Non-Small Cell Lung Cancer)) OR TS=(Nonsmall Cell Lung Cancer)

#2  (((((((((((TS=(Radiotherapy)) OR TS=(Radiotherapies )) OR TS=(Radiation Therapy )) OR TS=(Radiation Therapies )) OR TS=(Therapies, Radiation )) OR TS=(Therapy, Radiation)) OR TS=(Radiation Treatment )) OR TS=(Radiation Treatments )) OR TS=(Treatment, Radiation)) OR TS=(Radiotherapy, Targeted )) OR TS=(Radiotherapies Targeted)) OR TS=(Targeted Radiotherapies)
#3  (((((((((((((((((((((((((((((((((((((((((((((((((((((TS=(Brain Neoplasms)) OR TS=(Brain Neoplasm )) OR TS=(Neoplasm, Brain )) OR TS=(Neoplasms, Brain )) OR TS=(Brain Tumors )) OR TS=(Brain Tumor)) OR TS=(Tumor, Brain)) OR TS=(Benign Neoplasms, Brain)) OR TS=(Benign Neoplasm, Brain)) OR TS=(Brain Benign Neoplasm)) OR TS=(Brain Benign Neoplasms)) OR TS=(Neoplasms, Brain, Benign )) OR TS=(Brain Neoplasms, Benign )) OR TS=(Benign Brain Neoplasm)) OR TS=(Benign Brain Neoplasms )) OR TS=(Brain Neoplasm, Benign)) OR TS=(Neoplasms, Intracranial)) OR TS=(Intracranial Neoplasm)) OR TS=(Neoplasm, Intracranial )) OR TS=(Intracranial Neoplasms)) OR TS=(Brain Tumor, Primary )) OR TS=(Primary Brain Tumor )) OR TS=(Primary Brain Tumors )) OR TS=(Neoplasms, Brain, Primary)) OR TS=(Brain Neoplasm, Primary )) OR TS=(Primary Brain Neoplasms )) OR TS=(Brain Neoplasms, Primary )) OR TS=(Primary Brain Neoplasm)) OR TS=(Brain Tumor, Recurrent )) OR TS=(Brain Tumors, Recurrent )) OR TS=(Recurrent Brain Tumor )) OR TS=(Recurrent Brain Tumors)) OR TS=(Malignant Primary Brain Tumors )) OR TS=(Primary Malignant Brain Tumors  )) OR TS=(Malignant Primary Brain Neoplasms )) OR TS=(Primary Malignant Brain Neoplasms)) OR TS=(Brain Neoplasms, Malignant, Primary )) OR TS=(Brain Neoplasms, Primary Malignant )) OR TS=(Brain Metastases )) OR TS=(Brain Metastase)) OR TS=(Brain Cancer)) OR TS=( Brain Cancers )) OR TS=(Cancer, Brain )) OR TS=(Malignant Neoplasms, Brain)) OR TS=(Brain Malignant Neoplasm )) OR TS=(Brain Malignant Neoplasms )) OR TS=(Malignant Neoplasm, Brain )) OR TS=(Cancer of Brain)) OR TS=(Cancer of the Brain)) OR TS=(Neoplasms, Brain, Malignant )) OR TS=(Brain Neoplasms, Malignant)) OR TS=( Brain Neoplasm, Malignant)) OR TS=(Malignant Brain Neoplasm )) OR TS=(Malignant Brain Neoplasms)
#4   #1 AND #2 AND #3

Cochrane
#1 MeSH descriptor：º[Carcinoma，¬Non-small-Cell Lung] explode all trees
#2 MeSH descriptor：º[Radiotherapy] explode all trees
#3 MeSH descriptor：º[Brain Neoplasma] explode all trees

EMBASE
#1   'non small cell lung cancer'/exp
#2   'bronchial non small cell cancer'/exp OR 'bronchial non small cell carcinoma'/exp OR 'carcinoma, non-small-cell lung'/exp OR 'lung cancer, non small cell'/exp OR 'lung non small cell cancer'/exp OR 'lung non small cell carcinoma'/exp OR 'non oat cell lung cancer'/exp OR 'non small cell bronchial cancer'/exp OR 'non small cell cancer, lung'/exp OR 'non small cell lung carcinoma'/exp OR 'non small cell pulmonary cancer'/exp OR 'non small cell pulmonary carcinoma'/exp OR 'non squamous nsclc'/exp OR 'non-oat cell lung cancer'/exp OR 'non-small-cell lung carcinoma'/exp OR 'nonsmall cell carcinoma of the lung'/exp OR 'nonsmall cell lung cancer'/exp OR 'nonsmall cell lung carcinoma'/exp OR 'pulmonary non small cell cancer'/exp OR 'pulmonary non small cell carcinoma'/exp OR 'non small cell lung cancer'/exp
#3   #1 OR #2
#4   'radiotherapy'/exp OR 'radiotherapy'
#5   'bioradiant therapy':ab,ti OR 'bucky irradiation':ab,ti OR 'bucky radiation':ab,ti OR 'bucky radiotherapy':ab,ti OR 'bucky ray':ab,ti OR 'bucky ray radiation':ab,ti OR 'bucky therapy':ab,ti OR 'fractionated radiotherapy':ab,ti OR 'hemibody irradiation':ab,ti OR 'hypophysectomy, radiation':ab,ti OR 'hypophysis irradiation':ab,ti OR 'hypophysis radiation':ab,ti OR 'irradiation therapy':ab,ti OR 'irradiation treatment':ab,ti OR 'irradiation, hypophysis':ab,ti OR 'lymphatic irradiation':ab,ti OR 'pituitary irradiation':ab,ti OR 'radiation beam centration':ab,ti OR 'radiation repair':ab,ti OR 'radiation therapy':ab,ti OR 'radiation treatment':ab,ti OR 'radio therapy':ab,ti OR 'radio treatment':ab,ti OR 'radiohypophysectomy':ab,ti OR 'radiology, therapeutic':ab,ti OR 'radiotreatment':ab,ti OR 'roentgen irradiation, therapeutic':ab,ti OR 'roentgen therapy':ab,ti OR 'roentgen treatment':ab,ti OR 'rontgen therapy':ab,ti OR 'therapeutic radiology':ab,ti OR 'therapy, irradiation':ab,ti OR 'therapy, radiation':ab,ti OR 'therapy, roentgen':ab,ti OR 'treatment, irradiation':ab,ti OR 'treatment, radiation':ab,ti OR 'treatment, roentgen':ab,ti OR 'x radiotherapy':ab,ti OR 'x ray therapy':ab,ti OR 'x ray treatment':ab,ti OR 'x-ray therapy'
#6   #4 OR #5
#7   #3 AND #6
#8   'brain metastasis'/exp
#9   'brain metastatic tumor':ab,ti OR 'brain metastatic tumour':ab,ti OR 'brain tumor metastasis':ab,ti OR 'brain tumour metastasis':ab,ti OR 'cerebral metastasis':ab,ti OR 'metastasis, brain':ab,ti OR 'brain metastasis'
#10   #8 OR #9
#11   #7 AND #10
#12   'randomized controlled trial'/exp OR 'controlled clinical trial'/exp OR randomized:ti,ab OR placebo:ti,ab OR 'drug therapy':lnk OR randomly:ti,ab OR trial:ti,ab OR groups:ti,ab
#13   'clinical article'/exp OR 'controlled study'/exp OR 'major clinical study'/exp OR 'prospective study'/exp OR 'cohort analysis'/exp OR 'cohort':ti,ab OR 'compared':ti,ab OR 'groups':ti,ab OR 'case control':ti,ab OR 'multivariate':ti,ab
#14   #12 OR #13
#15   #11 AND #14

Wanfang
1.FT =('肺Î癌©'+'非Ç小¡细¸胞û') AND FT =('脑Ô转ª移Æ') AND FT =('放Å疗Æ'+'放Å射ä治Î疗Æ')
2.AB=('肺Î癌©'+'非Ç小¡细¸胞û') AND AB =('脑Ô转ª移Æ')AND AB =('放Å疗Æ'+'放Å射ä治Î疗Æ')
3.TI=('肺Î癌©'+'非Ç小¡细¸胞û')AND TI =('脑Ô转ª移Æ')AND TI =('放Å疗Æ'+'放Å射ä治Î疗Æ')

CNKI
1.	FT =('肺Î癌©'+'非Ç小¡细¸胞û') AND FT =('脑Ô转ª移Æ') AND FT =('放Å疗Æ'+'放Å射ä治Î疗Æ')
2.	AB=('肺Î癌©'+'非Ç小¡细¸胞û') AND AB =('脑Ô转ª移Æ')AND AB =('放Å疗Æ'+'放Å射ä治Î疗Æ')
3.	TI=('肺Î癌©'+'非Ç小¡细¸胞û')AND TI =('脑Ô转ª移Æ')AND TI =('放Å疗Æ'+'放Å射ä治Î疗Æ')


Supplementary materials 2 sensitivity analysis


Results of sensitivity analysis showing the stability of the results in the included studies: a. iORR; b. iDCR; c. One-year Survival Rate; d. Incidence of adverse reactions; e. leukopenia; f. nausea and vomiting; g. diarrhea; h. Rash; I. myelosuppression.


Supplementary materials 3: PRISMA checklist
Section and Topic 	Item #	Checklist item 	
TITLE 	
Title 	1	This study is identified as a meta-analysis	
ABSTRACT 	
Abstract 	2	This summary includes the Background, methods, results and discussion	
INTRODUCTION 	
Rationale 	3	Described in the introduction	
Objectives 	4	Stated in the introduction	
METHODS 	
Eligibility criteria 	5	This paper provided a detailed description of the inclusion and exclusion criteria	
Information sources 	6	Specify all databases, registers, websites, organisations, reference lists and other sources searched or consulted to identify studies. Specify the date when each source was last searched or consulted.	
Search strategy	7	Present the full search strategies for all databases, registers and websites, including any filters and limits used.	
Selection process	8	The screening process is included in the data collection	
Data collection process 	9	We describe the data collection in detail	
Data items 	10a	We provide a detailed description of the outcome indicators	
	10b	List and define all other variables for which data were sought (e.g. participant and intervention characteristics, funding sources). Describe any assumptions made about any missing or unclear information.	
Study risk of bias assessment	11	We provide a detailed description of the risk assessment tool	
Effect measures 	12	In the methodology section, we made a specific introduction to the measurement of each outcome indicator	
Synthesis methods	13a	Describe the processes used to decide which studies were eligible for each synthesis (e.g. tabulating the study intervention characteristics and comparing against the planned groups for each synthesis (item #5)).	
	13b	Describe any methods required to prepare the data for presentation or synthesis, such as handling of missing summary statistics, or data conversions.	
	13c	Describe any methods used to tabulate or visually display results of individual studies and syntheses.	
	13d	Describe any methods used to synthesize results and provide a rationale for the choice(s). If meta-analysis was performed, describe the model(s), method(s) to identify the presence and extent of statistical heterogeneity, and software package(s) used.	
	13e	Describe any methods used to explore possible causes of heterogeneity among study results (e.g. subgroup analysis, meta-regression).	
	13f	Describe any sensitivity analyses conducted to assess robustness of the synthesized results.	
Reporting bias assessment	14	We introduced the method of measuring bias.	
Certainty assessment	15	We used Cochrane Risk of Bias (ROB) to evaluate the evidence for an outcome.	
RESULTS 	
Study selection 	16a	Describe the results of the search and selection process, from the number of records identified in the search to the number of studies included in the review, ideally using a flow diagram.	
	16b	Cite studies that might appear to meet the inclusion criteria, but which were excluded, and explain why they were excluded.	
Study characteristics 	17	Cite each included study and present its characteristics.	
Risk of bias in studies 	18	Present assessments of risk of bias for each included study.	
Results of individual studies 	19	For all outcomes, present, for each study: (a) summary statistics for each group (where appropriate) and (b) an effect estimate and its precision (e.g. confidence/credible interval), ideally using structured tables or plots.	
Results of syntheses	20a	For each synthesis, briefly summarise the characteristics and risk of bias among contributing studies.	
	20b	Present results of all statistical syntheses conducted. If meta-analysis was done, present for each the summary estimate and its precision (e.g. confidence/credible interval) and measures of statistical heterogeneity. If comparing groups, describe the direction of the effect.	
	20c	We conducted subgroup analysis to further study	
	20d	Present results of all sensitivity analyses conducted to assess the robustness of the synthesized results.	
Reporting biases	21	Present assessments of risk of bias due to missing results (arising from reporting biases) for each synthesis assessed.	
Certainty of evidence 	22	We used Cochrane Risk of Bias (ROB) to evaluate the evidence for an outcome.	
DISCUSSION 	
Discussion 	23a	Provide a general interpretation of the results in the context of other evidence.	
	23b	Discuss any limitations of the evidence included in the review.	
	23c	Discuss any limitations of the review processes used.	
